# Utilizing the Dog Genome in the Search for Novel Candidate Genes Involved in Glioma Development—Genome Wide Association Mapping followed by Targeted Massive Parallel Sequencing Identifies a Strongly Associated Locus

**DOI:** 10.1371/journal.pgen.1006000

**Published:** 2016-05-12

**Authors:** Katarina Truvé, Peter Dickinson, Anqi Xiong, Daniel York, Kartika Jayashankar, Gerli Pielberg, Michele Koltookian, Eva Murén, Hans-Henrik Fuxelius, Holger Weishaupt, Fredrik J. Swartling, Göran Andersson, Åke Hedhammar, Erik Bongcam-Rudloff, Karin Forsberg-Nilsson, Danika Bannasch, Kerstin Lindblad-Toh

**Affiliations:** 1 Department of Animal Breeding and Genetics, Swedish University of Agricultural Sciences, Uppsala, Sweden; 2 Bioinformatics Core Facility, Sahlgrenska Academy, University of Gothenburg, Gothenburg, Sweden; 3 Department of Surgical and Radiological Sciences, School of Veterinary Medicine, University of California Davis, Davis, California, United States of America; 4 Department of Immunology, Genetics and Pathology, Science for Life Laboratory, Uppsala University, Uppsala, Sweden; 5 Department of Population Health and Reproduction, School of Veterinary Medicine, University of California Davis, Davis, California, United States of America; 6 Science for Life Laboratory, Department of Medical Biochemistry and Microbiology, Uppsala University, Uppsala, Sweden; 7 Broad Institute of Harvard and Massachusetts Institute of Technology (MIT), Cambridge, Massachusetts, United States of America; 8 Department of Clinical Sciences, Swedish University of Agricultural Sciences, Uppsala, Sweden; National Cancer Institute, UNITED STATES

## Abstract

Gliomas are the most common form of malignant primary brain tumors in humans and second most common in dogs, occurring with similar frequencies in both species. Dogs are valuable spontaneous models of human complex diseases including cancers and may provide insight into disease susceptibility and oncogenesis. Several brachycephalic breeds such as Boxer, Bulldog and Boston Terrier have an elevated risk of developing glioma, but others, including Pug and Pekingese, are not at higher risk. To identify glioma-associated genetic susceptibility factors, an across-breed genome-wide association study (GWAS) was performed on 39 dog glioma cases and 141 controls from 25 dog breeds, identifying a genome-wide significant locus on canine chromosome (CFA) 26 (p = 2.8 x 10^−8^). Targeted re-sequencing of the 3.4 Mb candidate region was performed, followed by genotyping of the 56 SNVs that best fit the association pattern between the re-sequenced cases and controls. We identified three candidate genes that were highly associated with glioma susceptibility: *CAMKK2*, *P2RX7* and *DENR*. *CAMKK2* showed reduced expression in both canine and human brain tumors, and a non-synonymous variant in *P2RX7*, previously demonstrated to have a 50% decrease in receptor function, was also associated with disease. Thus, one or more of these genes appear to affect glioma susceptibility.

## Author Summary

Gliomas are devastating malignant brain tumors that are very rarely curable. Despite extensive research to define pathways and genes involved in the development of disease, there is still an urgent need to improve therapy. Some dog breeds have a considerable elevated risk of glioma, making the dog a suitable model for locating genes potentially of importance also for development of human glioma. In this study we defined a genomic region strongly associated with glioma in dogs. We also showed that this genomic region had likely been under selection in the dog breeds with the highest risk of developing glioma. Sometimes selection for breed specific traits results in amplification of disease causing mutations together with the variant selected for. We located three candidate genes in the identified region: *CAMKK2*, *P2RX7* and *DENR*. We performed further functional studies to evaluate the potential role of these genes in both canine and human glioma. By comparing normal and tumor tissue we could show that two of the genes—*CAMKK2* and *P2RX7*, were affected at the level of gene expression and protein structure, respectively. We propose that further investigation of all three genes could be of interest with potential benefit to both dog and human.

## Introduction

Gliomas are the most common form of malignant primary brain tumors in humans, characterized by rapid growth and the invasion of neoplastic cells into healthy brain. Despite aggressive therapy, malignant gliomas are rarely curable. In the USA, the yearly mortality rates for primary malignant brain tumors are 5.6 and 3.7 per 100,000 in men and women, respectively [1]. To predict the biological behavior of the neoplasm and to standardize therapy regimes, brain tumors are classified and graded by the World Health Organization (WHO) according to location and histopathological appearance [2]. Mortality differs significantly by histology and age [1], with 2 year survival rates of less than 15% for the most aggressive and most common histological subtype, glioblastoma (GBM) [3]. Studies of syndromes and familial aggregation have suggested genetic susceptibility to gliomas, and although rare inherited mutations account for only few cases they are important for identifying pathways for gliomagenesis according to Brain Tumor Epidemiology Consortium [1]

Spontaneous gliomas in dogs are usually classified and graded using the human WHO criteria, [2] and have striking similarities to their human tumor counterparts at the biological and imaging levels [4,5,6,7,8]. Central nervous system tumors occur in dogs at an incidence of around 15 per 100,000 animals or 2–4% of necropsy cases, with gliomas representing approximately 35% of all CNS primary tumors [5,6,9]. This is similar to human patients where gliomas represent 24% of all primary CNS tumors with an incidence of approximately 20 per 100,000 [3] Extensive analysis of gliomas in humans has defined commonly disrupted pathways involving the receptor tyrosine kinases/PI3K/RAS, TP53 and RB1 pathways [10,11]. Defining molecular subclasses of glioma will likely guide future therapeutic and prognostic stratification [10,12]. Although the frequency of specific glioma subtypes varies between humans and dogs, with humans having more high grade glioblastomas and dogs having more high grade oligodendrogliomas [3,4,5], ppreliminary analysis suggests that the same key pathway abnormalities are also present in gliomas in dogs [4,13,14,15,16].

Dogs are excellent spontaneous models of human complex diseases including cancers, by sharing both genetic and environmental factors. In addition, the recent breed creation events, resulting in certain diseases becoming overrepresented in specific breeds, have made disease gene mapping easier than in human populations. Genome wide association studies in dogs have been highly successful owing to long-range linkage disequilibrium (LD), available SNP genotyping tools and a robust genome assembly [17,18]. There are many examples of successful GWAS in dogs for Mendelian traits [17,19,20,21,22,23] as well as for more complex traits [24,25,26,27,28]. In addition, across-breed mapping studies by us and others have successfully identified biologically relevant loci for several traits and related diseases that are fixed within breeds [29,30,31,32].

Some brachycephalic dog breeds have been reported to have a considerable elevated risk of glioma, such as Boxer (relative risk ~23), Bulldog and Boston Terrier (relative risk ~5) [6,33]. Even though all brachycephalic breeds are likely to share a common major mutation that causes this phenotype [30] all brachycephalic breeds are not reported to be at higher risk of developing glioma. A study using neighbor-joining trees on genome wide SNP-data for dog breeds has shown that Boxer, English Bulldog, Boston Terrier and French Bulldog are closely related [34] and likely to share a recent common ancestor. Other brachycephalic breeds such as Pug and Pekingese are closely related to each other [34,35] but not as recently related to the high-risk glioma breeds. We hypothesized that genetic risk factors for glioma have been segregating in an “ancestral bulldog” line. Because of the extraordinary high risk of all types of glioma in the Boxer, we hypothesized that genetic risk factors might be in a nearly fixed region of the Boxer genome. Since it has been reported that the risk for glial tumors in dogs increases with age until 10–14 years [33], and the possibility of a nearly fixed risk locus, we concluded that the traditional affected—nonaffected within breed association mapping approach would not be suitable. Instead we hypothesized that some genetic risk factors for glioma might be shared between breeds and given the suggested recent relationship for some of the breeds at high risk, an across-breed mapping approach comparing glioma-cases from several breeds to controls from several breeds could identify these risk factors. Given the similarities between human and canine gliomas at the histological and genetic level, we hypothesized that this approach could identify genes or pathways that may also be relevant for human glioma.

## Results

To search for glioma risk factors we performed an across-breed GWAS identifying a major risk locus on CFA 26. This region was further studied using targeted re-sequencing and validating genotyping, followed by gene expression studies of the identified candidate genes in both dogs and humans.

### Tumor type frequency assessment and sample collection

During the period of 1987–2013 a total of 228 gliomas were histologically diagnosed at UC Davis. Among these, oligodendrogliomas were overrepresented in English Bulldog, French Bulldog, Boston Terrier, and Boxers; astrocytomas were overrepresented in Boston Terrier, Boxer, Jack Russell Terrier and Pit Bull Terrier. When all gliomas including mixed oligoastrocytomas were evaluated, Mastiffs also had an overrepresentation of tumors (Table 1).

**Table 1:** Significantly overrepresented breeds for specific tumor types. Dog breeds significantly overrepresented in histologically diagnosed gliomas compared to representation in the general hospital population. Percentage of specific gliomas in each breed from the total (228) is shown. All = Oligodendrogliomas, Astrocytomas and mixed oligoastrocytomas combined. NS = tumor type not statistically significantly overrepresented in the breed. Significance is based on a likelihood Chi-square association test.

Breed	Hospital pop %	Oligodendrogliomas% *n* = 110	Astrocytomas% *n* = 88	All glioma% *n* = 228	Significance
**Boxer**	1.8	31.8	20.5	28.5	All < 0.00001
**English Bulldog**	0.7	9.1	NS	6.1	All < 0.00001
**French Bulldog**	0.2	10.9	NS	5.3	All < 0.00001
**Boston Terrier**	0.7	7.3	5.7	6.1	< 0.00001, 0.0002, < 0.00001
**Jack Russell Terrier**	0.9	NS	4.5	NS	0.04
**Pit Bull Terrier**	0.7	NS	4.5	3.5	0.007, 0.006
**Mastiff**	0.5	NS	NS	2.6	0.007

### Across-breed genome-wide association mapping

We performed genome-wide association mapping, using 39 glioma cases diagnosed with varying types and grades of glioma and 141 controls comprising 25 different dog breeds and 4 mixed breed dogs (Table 2, S1 Table). The controls were selected to represent several breeds with a few individuals from each breed to maximize the power of the study, and to decrease bias due to population stratification.

**Table 2:** Samples from different breeds included in glioma GWAS.

Cases:		Controls:	
Australian Shepherd	2	Australian Shepherd	1
Boston Terrier	2	Beagle	11
Boxer	9	Australian Cattle Dog	1
English Bulldog	2	Cocker Spaniel	1
Australian Cattle Dog	1	Dachshund	13
French Bulldog	2	German Shepherd	12
Jack Russell Terrier	1	Greyhound	11
Keeshond/Border Collie	1	Jack Russell Terrier	12
Labrador/Border Collie mix	1	Japanese Chin	1
Labrador Retriever	7	Labrador Retriever	12
Mastiff	2	Mastiff	1
Husky /Labrador Retriever mix	1	Nova Scotia Duck Tolling Retriever	14
Pit Bull mix	3	Pekingese	1
Rhodesian Ridgeback	1	Poodle	12
Soft-coated Wheaten Terrier	1	Pug	13
American Staffordshire Terrier	2	Rhodesian Ridgeback	3
West Highland White Terrier	1	Soft-coated Wheaten Terrier	1
**Total**	39	Shar Pei	12
		Weimaraner	8
		West Highland White Terrier	1
		**Total**	141

**Fig 1:**
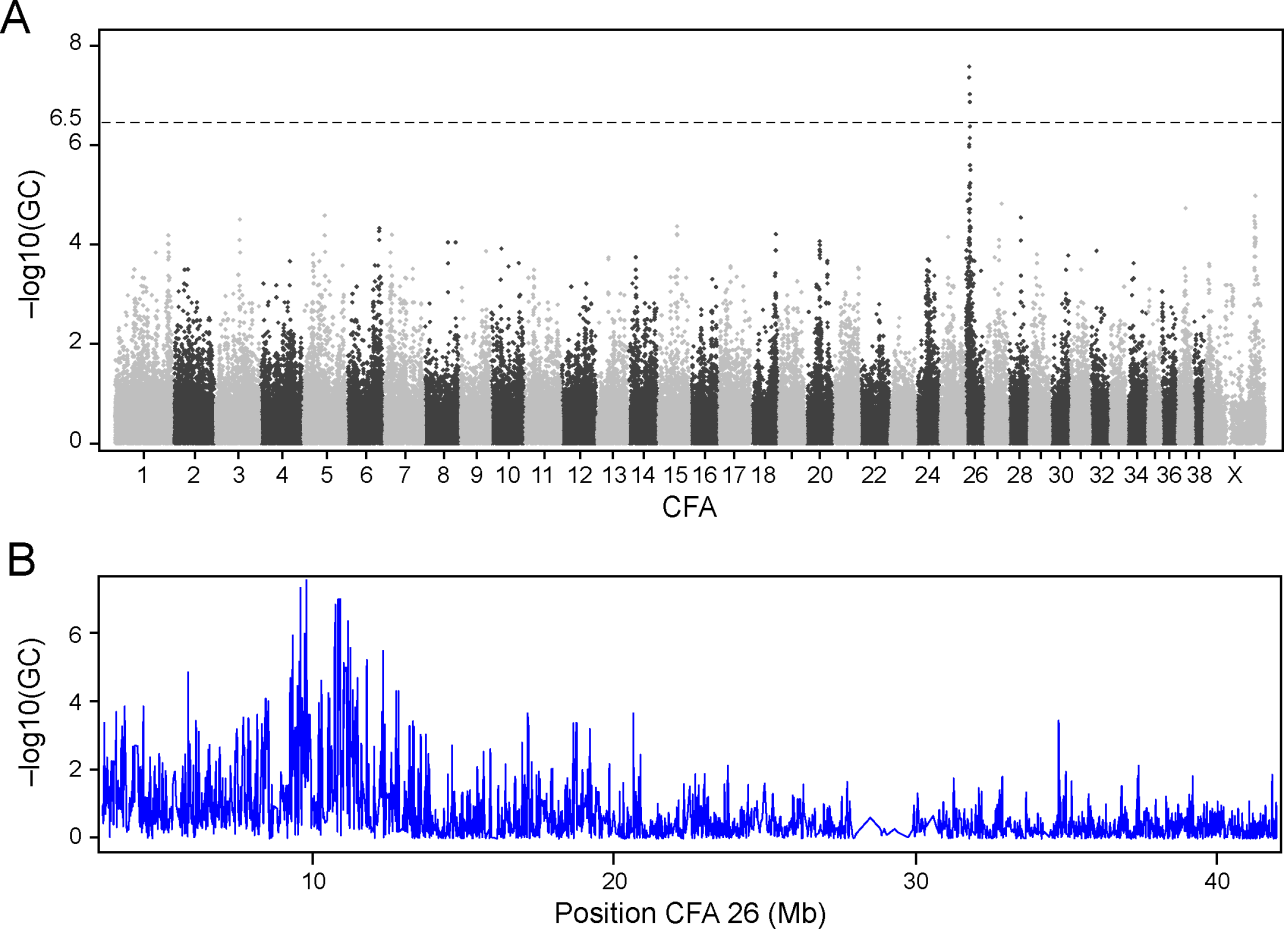
Across-breed GWAS for glioma identified a strongly associated locus on chromosome 26. Log p-values on the y-axis are adjusted for stratification by genomic control (GC). A significantly associated locus was found on CFA 26 (A) spanning the region of CFA 26:8.5–12 Mb (B). Dashed line at log p-value 6.5 corresponds to p-value 0.05 after Bonferroni correction (A).

Based on the uneven number of cases and controls between breeds, we expected a presence of population structure and applied the genomic control (GC) option in PLINK [36] to correct for population structure. After GC correction, a major locus on CFA 26 (top SNP = 9,780,187, CanFam2.0, p_raw_ = 5.47x 10^−38^, p_gc_ = 2.77 x 10^−8^, Fig 1A) was identified. Visual inspection of graph Fig 1B limits the associated region to ≈CFA 26: 8.5–12 Mb. (Five most associated SNPs in S2 Table). Bonferroni correction was applied to calculate a significance threshold adjusted for multiple comparisons.

Since the Boxer breed had the largest number of cases (n = 9, 23%), a separate association analysis was performed excluding the representatives of this breed from the dataset, in order to check for an excess impact from this population. The same region on CFA 26 was identified (p_raw_ = 9.7 x 10^−28^). After correction by genomic control the association was in fact stronger after removal of the Boxers (p_gc_ = 9.1 x 10^−11^, S2 Fig), reflecting a lower genomic inflation. A Quantile Quantile (QQ) plot showed low remaining inflation, with p-values starting to slightly deviate from the expected curve at–logP of 2, but with a much sharper deviation at expected significance level of–logP 6.5 (S3 Fig).

### Signature of selective sweep in ancestral bulldog line

**Fig 2:**
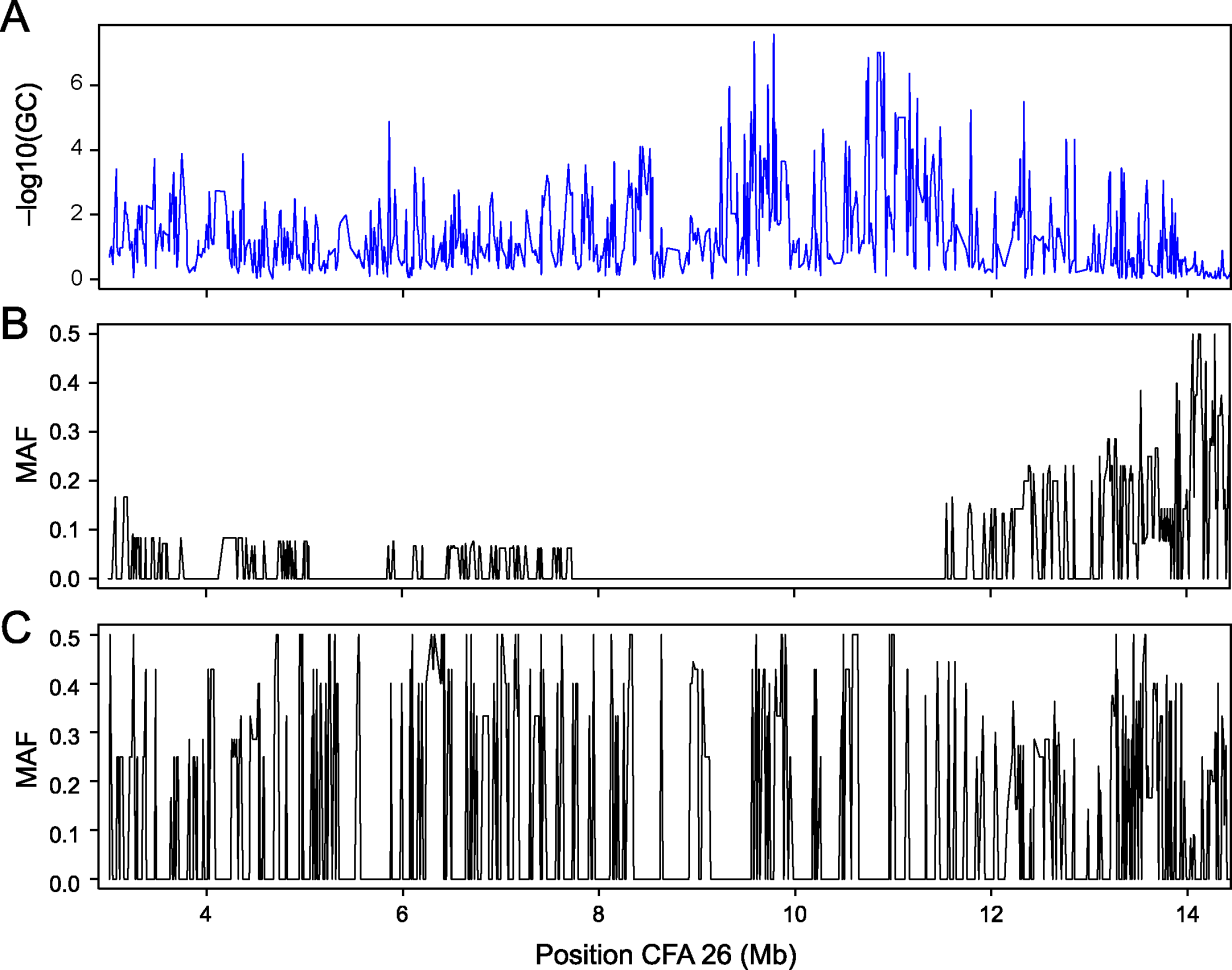
Glioma-associated region co-localizes with a selective sweep. A view of the most associated region for glioma (A) and inspection of minor allele frequencies shows that low minor allele frequencies (indicative of selection) in breeds related to an ancestral Bulldog (B) co-localize with the associated region. No comparable signs of selection are present in the Pugs (C).

We calculated pairwise identity-by-state genetic distances between all samples using PLINK [36], and then constructed a phylogenetic tree using the software Phylip. The results (S1 Fig) supported previous studies [34,35] reporting a close relationship for the four breeds: Boxer, English Bulldog, French Bulldog and Boston Terrier. Reduced heterozygosity in the dog genome can occur because of genetic drift or selection. Homozygous blocks longer than 1 Mb have been proposed to be more likely to have arisen through selection than drift [29]. We examined the minor allele frequency (MAF) on CFA 26 to check for signs of selection in the region associated with glioma. The MAF for the four most recently related breeds of the ancestral bulldog line was compared to the MAF for Pugs (Fig 2). A common completely homozygous region in the ancestral Bulldog line spanning ~ 4 Mb (Fig 2B) including the glioma-associated region (Fig 2A) was identified. An additional ~ 8 Mb flanking this region showed reduced heterozygosity. This can be interpreted as a likely selective sweep having taken place in the ancestral Bulldog line leaving traces behind in descending modern breeds. Since it is known that brachycephaly is a trait that has been under selection we made a comparison to Pugs to investigate if this was a common sweep in brachycephalic breeds. Pugs showed no comparable homozygosity or signs of selection in this region (Fig 2C), supporting their difference in ancestry. In this study the sweep was identified using dogs with glioma. Other reports support that this region has been under selection in a normal cohort of Boxers [37] and English Bulldogs [29]. This locus was also identified as the second most associated in our previous study identifying a major locus for brachycephaly [30], which could be explained by its presence in only a subpopulation of brachycephalic breeds.

### Targeted re-sequencing of associated regions

In order to find putative genetic risk factors for glioma we performed targeted re-sequencing using NimbleGen capture followed by Illumina sequencing. In total 3.4 Mb were re-sequenced spanning 8.5–11.9 Mb on CFA26. The sequencing was performed in two experiments. In the first experiment the most homozygous region on CFA26 (8.5–9.2 Mb and 10.8–11.9 Mb) was sequenced in six dogs; three brachycephalic (one Pug and two Boxers) and three control breeds (Dachshund, Welsh Corgi, and Basset Hound). The focus was on identifying differences between brachycephalic and non-brachycephalic dogs. In the second experiment the target region on CFA26 was extended. Four dogs were sequenced: three brachycephalic dogs diagnosed with glioma and one Dachshund as a control. The two experiments were analyzed together to identify potential risk variants for glioma. In addition a pool of dog-breeds from a whole genome sequencing project were utilized as controls (See Materials and Methods).

In total, 6,957 Single nucleotide variants (SNV) were identified in the re-sequenced region when mapping the reads against the Boxer reference genome sequence [18]. Out of these, 490 SNVs were located within conserved elements (+- 5bp). In addition, we identified one large structural polymorphism. An insertion (in the Boxer reference genome sequence) of ~2,200 bp on CFA26: 9,550,700–9,552,900 was identified in the glioma-associated region by showing a total lack of coverage for the Dachshund, but normal coverage for the brachycephalic/glioma individuals.

Candidate variants were selected from the re-sequencing data for a replicate study to evaluate disease association. Since a mutation in a region that is conserved across species is more likely to have a function, we used SEQscoring [38] in the selection process to score the SNVs by conservation and to rank them according to differences between cases and controls.

### Evaluation of identified candidate variants

A selection of 56 candidate SNVs were genotyped in a total of 168 dogs using Sequenom. Association tests were performed in three different ways using PLINK [36]. In the first analysis, all dogs were included with their original status as cases (n = 34) or controls (n = 134). After the first analysis we calculated the allele frequency for all breeds separately for the two most significant SNVs (S8 Table). We concluded that Boxer, English Bulldog, and Boston Terrier seemed fixed at these positions (frequency 0.93–1.00 for risk alleles). In a second analysis we removed all samples from these fixed breeds to investigate if the same genes would be the most associated in the remaining 21 cases and 113 controls. In a third analysis we instead assumed that if the samples from the high risk breeds that seemed fixed actually all carried the risk factor then it would make sense to perform a test where we assign all these samples a status as cases (n = 55)

**Fig 3:**
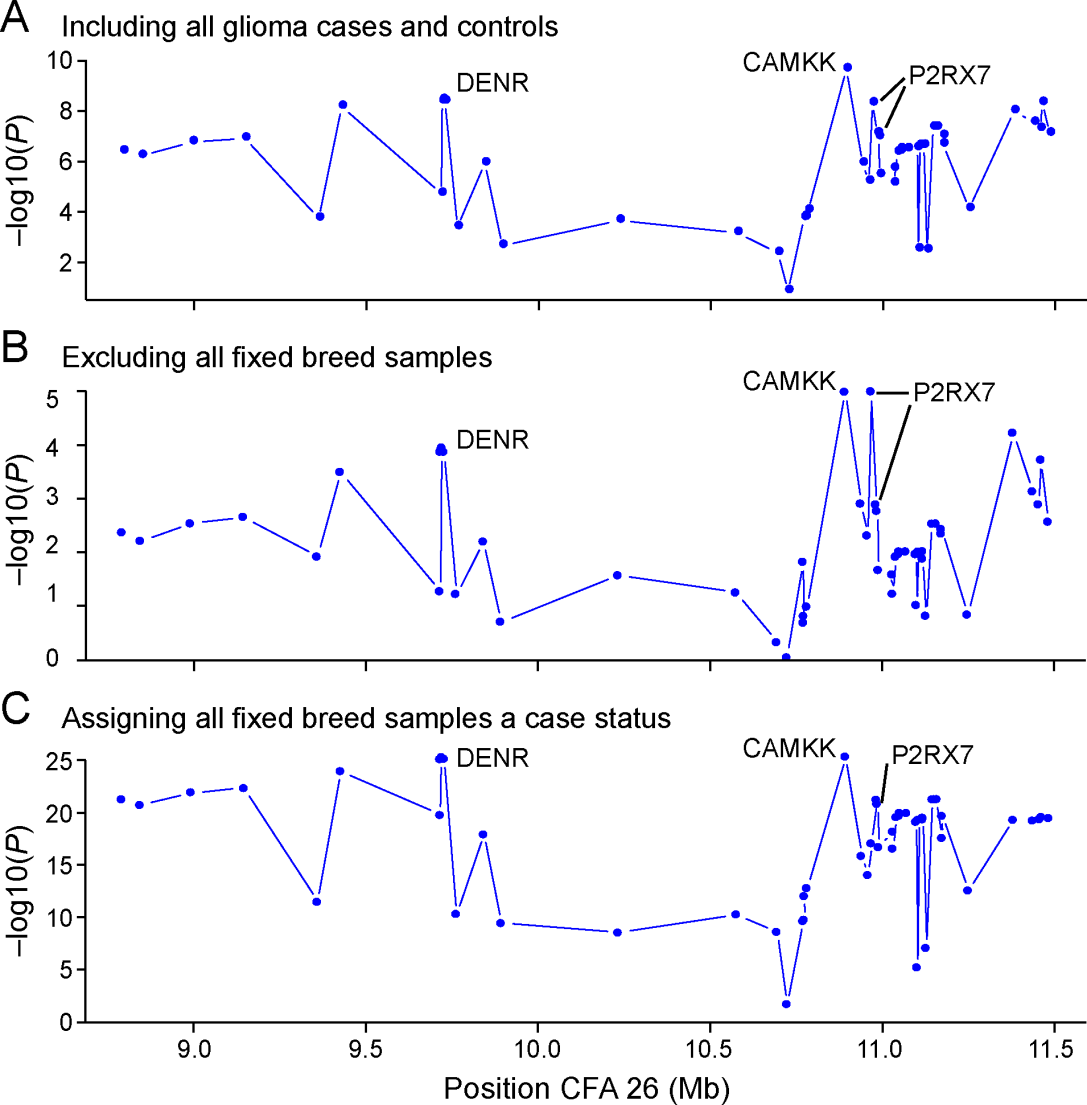
Fine-mapping of candidate SNVs shows the strongest association within the *CAMKK2* gene. Genotyping of 56 candidate SNVs showed the strongest association within an intron of *CAMKK2* for three tests: Including all glioma cases and controls (A), excluding all fixed breed samples (B), and assigning all fixed breed samples a status as cases (C). Second strongest association was seen in an intron of *DENR*, and in the gene *P2RX7* where one SNV was non-synonymous coding.

In all three tests the most associated SNV was identified at position CFA26:10,893,462 located in an intron of the gene Calcium/calmodulin-dependent protein kinase kinase 2 (*CAMKK2*, alias *CAMKK*, *CAMKKB*) (Fig 3A, 3B and 3C) (with p-values p = 3.67e-09 (A), 1.04e-05 (B), 4.75e-26 (C). The second most associated SNV was identified at position CFA26:9,722,698 in the first (p = 2.98e-08) and the third test (p = 5.04e-26), located in an intron of the gene Density-regulated protein *(DENR)*. The position was conserved among placental mammals [39]. In the second test the second most associated SNV was identified at position CFA26:10,969,340 (p = 1.05e-05) located in an intron of the gene Purinergic receptor P2X, ligand gated ion channel *7 (P2RX7)*. This position was not conserved, but another position located in the third exon of this gene is conserved and causes a non-synonymous codon change (CFA 26: 10,984,721). In the third test, the non-synonymous SNV in *P2RX7* was more associated (p = 6.70e-22) than the intronic SNV (CFA26:10,969,340 p = 8.89e-18) in this gene. This non-synonymous polymorphism causes an amino acid change from phenylalanine (F) to leucine (L) (p.Phe103Leu). The exchanged amino acid is involved in the extracellular loop of the trans-membrane protein P2RX7. We selected the three SNVs: CFA26:10,893,462, CFA26:9,722,698 and CFA26:10,984,721 in the three genes *CAMKK*, *DENR* and *P2RX7* for further analysis. (P-values reported in the evaluation of SNPs are obtained from basic Chi-square tests with no further correction.)

Individual genotypes for the candidate SNVs can be found in S5 Table. In addition, a candidate structural variant was evaluated for disease association. The ~2,200 bp insertion on CFA26: 9,550,700–9,552,900 was genotyped in 147 dogs (32 cases and 115 controls). The insertion was shown to be fairly common in several breeds, and was much less associated (p = 0.001, S5 Table) with glioma than evaluated SNVs in the region.

### Extended genotyping in breeds segregating for the identified variants

To further investigate if the identified variants were truly associated with glioma, we selected six breeds that were segregating for the identified variants. To better avoid confounding breed effects, we added more healthy controls from the same breeds. In total we genotyped 15 cases and 119 controls (Table 3) for three the selected SNVs, located in *CAMKK*, *DENR and P2RX7*.

**Table 3:** Genotyping in breeds segregating with the disease.

Samples			Association				
Breeds	Cases	Controls	Chr	Position	Gene	P-value	Odds Ratio
Australian Cattle Dog	1	18	26	10,893,462	CAMKK	4.37E-09	8.7
Labrador Retriever	6	21	26	9,722,698	DENR	1.21E-07	7.104
Mastiff	2	19	26	10,984,721	P2RX7	2.51E-08	7.846
Pit Bull Terrier	3	25					
Staffordshire Terrier	2	19					
West Highland White Terrier	1	17					
Total	15	119					

The odds ratios combined across all six breeds were *CAMKK*: 8.7, *P2RX7*: 7.8, *DENR*: 7.1 Individual genotypes for the SNVs can be found in S9 Table.

### Expression analysis of candidate genes in normal and tumor dog tissue

**Fig 4:**
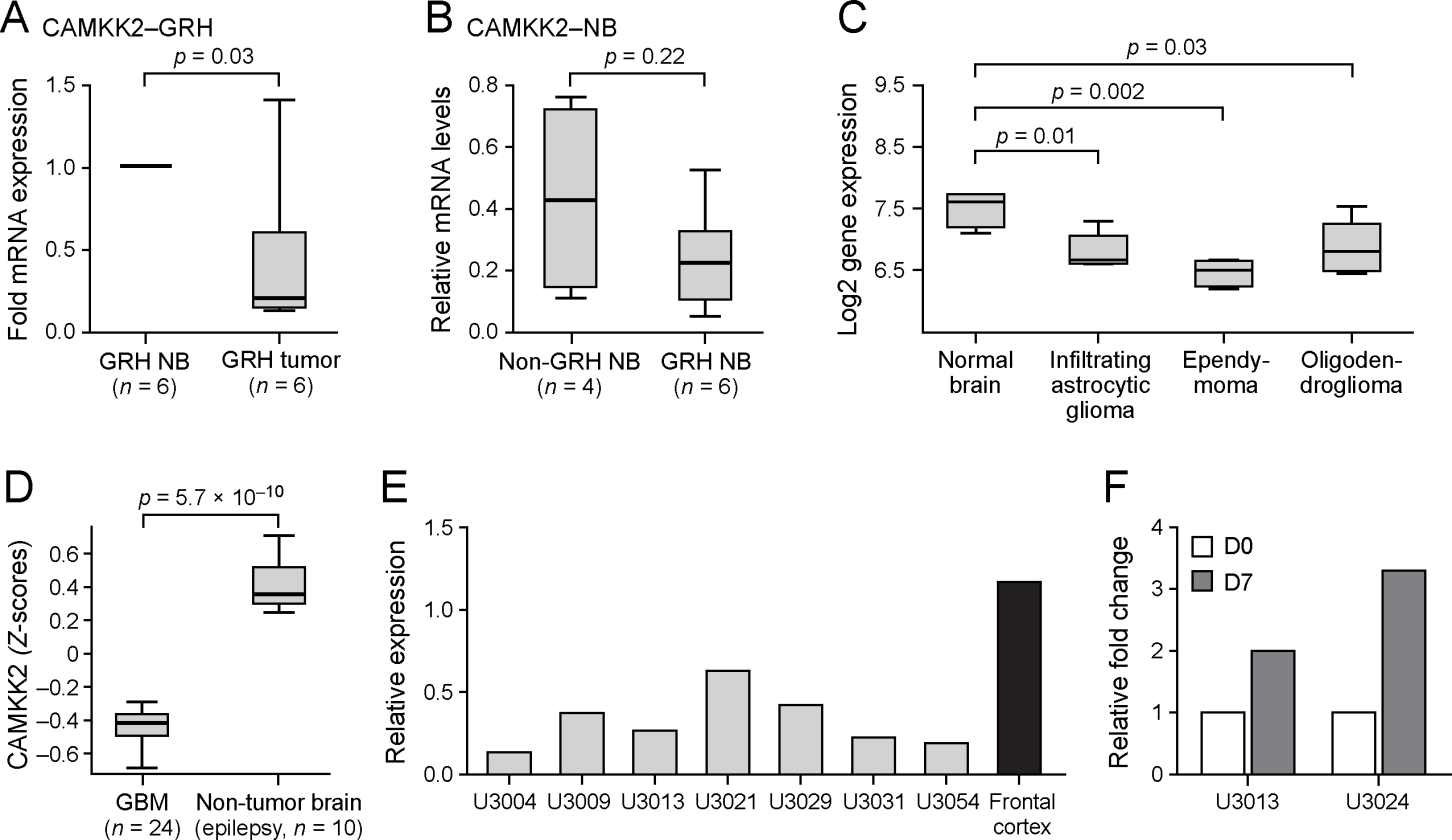
Expression changes of *CAMKK2* in canine and human brain tumors. Distributions of expression values in the different groups are depicted as box-and-whisker-plots, with the median of expression represented by horizontal lines inside each box, lower and upper box borders indicating 25th and 75th percentiles, respectively, and regions between whiskers including all non-outlier values. (A) Quantitative RT-PCR comparing matched normal brain (nominally 1.0) to tumor tissue in dogs with the glioma risk haplotype (GRH) and (B) comparing normal cerebrum from GRH and non GRH tissue. Significantly decreased *CAMKK2* mRNA expression is seen in GRH tumors. **(**C) Human expression data was obtained from a study by Liu and colleagues [40]. Comparing to normal brain (n = 4), mRNA expression level of *CAMKK2* is significantly lower in astrocytic glioma (n = 5), ependymoma (n = 4), and oligodendroglioma (n = 5). (D) Another set of human expression data was obtained from the TCGA Research Network and shows a significant lower expression in GBM patients (n = 24) compared to epileptic normal brain (n = 10). (E) Quantification of Western blot of CAMKK2 in human patient-derived GBM cell lines and normal adult brain lysate. Comparing to adult brain, GBM cell lines exhibit a lower level of CAMKK2. (F) When induced to differentiation with serum, *CAMKK2* mRNA levels increase in patient-derived GBM cells between day 0 (D0) and day 7 (D7). This experiment has been repeated twice.

To evaluate the effect of potential candidate variants on the expression of genes in the region of interest we performed quantitative PCR experiments on tumor and matched normal cerebral samples from the same animals. A reduced expression of *CAMKK2* was seen in tumor versus normal samples (n = 6, p = 0.03) in dogs with the three SNPs glioma risk haplotype (GRH) (Fig 4A).

**Fig 5:**
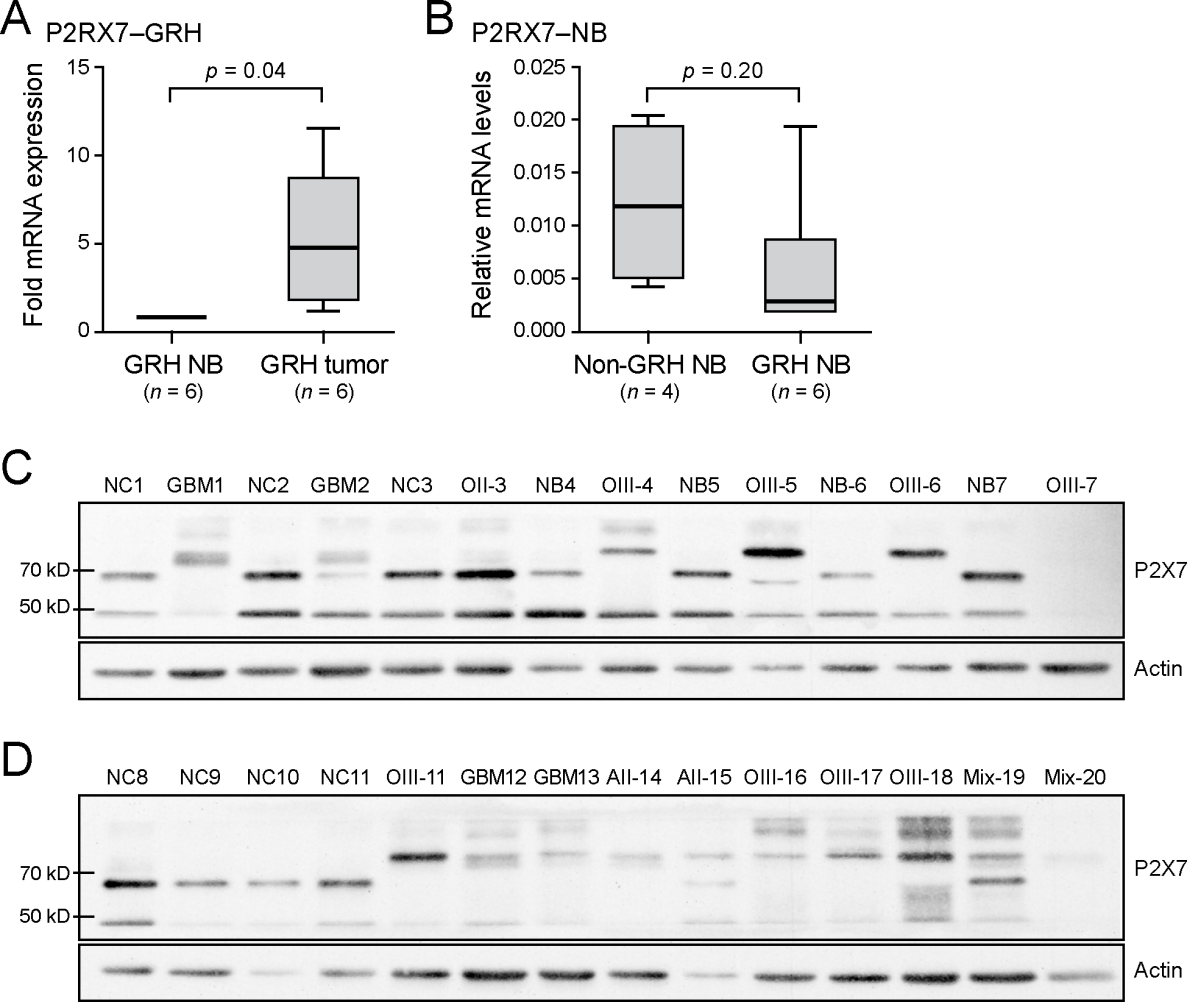
Expression of *P2RX7* in canine gliomas. (A) Quantitative RT-PCR comparing matched GRH normal brain (nominally 1.0) to GRH tumor tissue and (B) comparing normal cerebrum from GRH and non-GRH tissue. *P2RX7* mRNA expression using primers recognizing the majority of known splice variants is significantly elevated in gliomas (A). Western blotting of P2RX7 in matched normal cerebrum and tumors (C) and non-matched normal and tumor (D) showing consistently higher molecular weight bands for gliomas compared to normal brain. NC = normal cerebrum, GBM = glioblastoma, O = oligodendroglioma, A = astrocytoma, NB = normal brain (cerebrum) matched to tumor sample, Mix = Oligoastrocytoma.

The risk haplotype also appeared to induce a non-significant ~2-fold lower expression in normal tissue in dogs with the risk haplotype (n = 6) versus dogs without the risk haplotype (n = 4, p = 0.22, Fig 4B). No significant changes were seen for *DENR*. *P2RX7* showed an increased mRNA expression in tumors versus normal tissue (n = 6, p = 0.04) in dogs with the risk haplotype (Fig 5A) while there was no significant difference in normal tissue in dogs with the risk haplotype (n = 6) versus dogs without the risk haplotype (n = 4, p = 0.20, Fig 5B). P2RX7 protein expression was assessed in matched and unmatched normal cerebrum and tumors (Fig 5C and 5D) by western blotting and was detected in all canine normal brain samples and in 16/17 glioma samples. Major bands previously reported to represent glycosylated (~75kD) and un-glycosylated (~60kD) protein were seen, as well as a consistent band at around 50kD that was present in all samples. No significant difference in total protein levels was detected. However, normal brain samples consistently expressed only the 60kD band, while the majority of tumor samples expressed the 75kD and other higher molecular weight bands with minimal expression of the 60kD band.

### Expression of candidate genes in human glioma and glioblastoma cell lines

Based on the expression changes seen in canine tumors, we examined human high-grade gliomas for similar expression changes. Analysis of an available array dataset [40] revealed a significantly lower mRNA expression of *CAMKK2* in infiltrating astrocytic tumors (n = 5, p = 0.01), ependymoma (n = 4, p = 0.002), and oligodendroglioma (n = 5, p = 0.03) compared to normal brain tissue (n = 4, Fig 4C). Using a subset of data from the Cancer Genome Atlas (TCGA) [11] consisting of mRNA expression data of surgical specimens from 24 glioblastoma patients, and 10 non-tumor control brains (epilepsy resections), we confirmed the lower mRNA expression of *CAMKK2* in high-grade glioma (Fig 4D). We found no significant difference for *DENR* or *P2RX7* expression in these same dataset (S4 Fig). Furthermore, we analyzed the levels of CAMKK2 expression in seven human glioblastoma patient-derived cell lines, maintained in neural stem cell medium under serum-free conditions [41] and found that all tumors showed 20–60% lower level of expression of CAMKK2 protein compared to normal frontal cortex (Fig 4E). Because CAMKK2 expression is associated with a mature cellular phenotype [42] we next treated two of the glioblastoma cell lines with 5% serum for seven days to induce their differentiation. For both cell lines *CAMKK2* mRNA expression increased 2–3 fold with differentiation (Fig 4F), suggesting that *CAMKK2* expression is reduced in the more stem cell like tumor cells. There was a trend towards lower DENR protein expression in GBM compared to normal brain, but P2RX7 showed no conclusive difference in protein expression.

## Discussion

In this study we successfully identified a locus strongly associated with glioma across several dog breeds. Given that the power of canine disease gene mapping typically results from the enrichment of genetic risk factors for specific diseases within a breed, and that disease mapping tools have been designed for within breed mapping, this means that across-breed mapping studies have been challenging so far. Here we took advantage of the fact that glioma has an increased frequency in several related brachycephalic breeds derived from an ancestral Bulldog, the Boxer, English Bulldog, French Bulldog and the Boston Terrier. The glioma-associated region resides in a region showing a sweep in these brachycephalic dogs, suggesting that the across-breed mapping is made feasible by the long selected haplotype in this region. We hypothesize that genetic risk factors for glioma have been enriched in these breeds related to an ancestral Bulldog and have either hitch-hiked with a desirable trait during selective breeding or are pleiotropic effects resulting from selected genetic variants. The risk allele was also present in six additional breeds affected with glioma (the Australian Cattle Dogs, West Highland White Terrier, Labrador Retriever, Mastiff, Pit Bull Terrier and the American Staffordshire Terrier) and conferred a significant risk within those breeds.

Re-sequencing and fine mapping of the region on CFA 26 associated with glioma identified three candidate genes; *DENR*, *CAMKK2* and *P2RX7* with the strongest associated SNV located within an intron of *CAMKK2*. Intriguingly, all three genes are potentially relevant candidate genes for cancer development and contain highly associated SNVs, offering the possibility that multiple variants contribute to disease at this extended locus.

The *CAMKK2* gene encodes a Ca^2+^/calmodulin-activated kinase, which is highly expressed in the adult brain [42]. Following an increase in intracellular Ca^2+^, CAMKK2 activates CAMKI, CAMKIV, Akt and AMP-activated protein kinase (AMPK) in a number of pathways [43,44]. It has been shown that elevated intracellular Ca^2+^ stimulates ERKs with a requirement for CAMKK2 acting through CAMKI and via RAS [45]. In our study, *CAMKK2* expression was significantly lower compared to normal brain in both canine and human gliomas, making it an attractive candidate gene for further investigations. CAMKK2 has been shown, together with CAMKIV, to be involved in cerebellar granule precursor migration and differentiation during normal development [42]. Since we found that differentiation of glioblastoma stem cells was correlated with a higher expression of *CAMKK2* we suggest that *CAMKK2* down regulation may render tumor cells more stem cell-like thereby increasing the aggressiveness of the tumor. It was recently reported that inhibition of CAMKK2 blocks migration of medulloblastoma via CAMKI, and CAMKK2 was proposed as a putative target to limit metastasis in this type of brain tumor [44]. On the other hand, CAMKK2 is a versatile activator of signaling pathways and in non-small cell lung cancer [46] its role in AMPK activation was proposed as a mechanism for tumor regression. Because normal brain, in both dogs and humans, express higher levels of *CAMKK2* than glioma, we propose that targeting CAMKK2 should involve its activation rather than inhibition.

The *DENR* gene encodes the Density-regulated protein, which acts together with the oncogene multiple copies in T-cell lymphoma-1 (*MCT-1*) in translation initiation [47]. Because DENR inactivation in Drosophila is lethal due to impaired histoblast proliferation, the DENR-MCT-1 complex was suggested to regulate translation of specific mRNAs, presumably from “cancer-relevant” genes, i.e. those involved in cell growth [48]. While no significant expression changes were seen in this study, DENR may be involved in a translational control system with a key role in supporting proliferation and tissue growth.

P2RX7 is a trimeric ligand-gated cation channel that mediates numerous downstream events following activation by extracellular adenosine 5'-triphosphate (ATP). The non-synonymous canine SNV associated with glioma in this study has recently been shown to result in approximately 50% decrease in P2RX7 function [49], and non-synonymous SNPs in *P2RX7* have been identified in an increasing number of human patients with a variety of conditions including cancer [50]. P2RX7 receptors are expressed in a wide variety of immune cells including microglia, the primary antigen-presenting cell of the central nervous system and P2RX7 function is important for IL-β release and downstream priming of IFNγ producing CD8+ T cells involved in adaptive immunity against tumors [51,52,53]. Abrogation of P2RX7 has been associated with decreased response to foreign material in graft versus host models, increased metastatic potential in human breast cancer and increased susceptibility to colon and epithelial cancers [51,54,55,56]. However, the role of P2RX7 in glioma is complex and expression has been shown to result in both suppression and an increase in glioma growth in a variety of models, suggesting additional effects beyond immune surveillance [53,57,58,59,60]. In addition to the higher *P2RX7* mRNA levels that we detected in dog glioma, we report an intriguing pattern of differently sized proteins occurring between tumor and non-tumor dog brain. It remains to be investigated how these relate to a potential glioma susceptibility mechanism.

In conclusion, this study identifies a locus associated with canine glioma that has likely been under selection in brachycephalic dog breeds related to the original Bulldog, where highly disease-associated SNVs are found in three neighboring candidate genes. Candidate functional consequences were observed for two of the genes suggesting that the glioma susceptibility may be conferred by multiple variants within this locus.

## Materials and Methods

### Selection of dogs for glioma GWAS

Blood samples were collected from canine patients of the University of California at Davis William R. Pritchard Veterinary Medical Teaching Hospital (VMTH). In total, 39 glioma cases of differing types and grades (as determined by a board certified veterinary pathologist) and 142 controls comprising 25 different dog breeds and 4 mixed breeds were collected for the GWAS (Table 1, S1 Table). The controls were selected to represent several breeds with a few individuals from each breed to maximize the power of the study, and to decrease bias due to population stratification. Frequency of glioma types within specific breeds was determined for all histologically diagnosed gliomas during the years 1987–2013. A likelihood ratio chi-square test was used to compare the presence or absence of individual tumor types with breed relative to the VMTH population for each breed. A p-value of <0.05 was used to define unusually large or small breed associations based on the distribution of breeds examined.

### Genome-wide SNP data

All samples were genotyped on the Illumina 170 K canine SNP array [29]. Association analysis was performed using the software package PLINK [36] calculating single marker chi-square association. Data quality control was performed to ensure a minor allele frequency (MAF) > 0.05 and call rate > 95% for both SNPs and individuals to be retained. One individual was removed because of low genotyping. After frequency and genotyping pruning, there were 143,007 SNPs left. The association calculations were further corrected for stratification by the use of genomic control (GC). PLINK was also used to format data for a QQ plot (S3 Fig) and MAF graphs (Fig 2). Significance level corresponding to p-value 0.05 after adjusting for 143 K test with Bonferroni correction was calculated and set to a log p-value of ≈6.5

To evaluate the relationship between breeds we calculated pairwise identity-by-state genetic distances between all samples using PLINK [36], and then constructed a phylogenetic tree using the Neighbor-Joining/UPGMA method of software Phylip (version 3.695, Joseph Felsenstein, University of Washington, Seattle).

### Targeted re-sequencing

Targeted massive parallel re-sequencing was performed in two experiments. In the first experiment a total of ≈1.8 Mb was targeted (CFA26: 8,534,645–9,176,011, CFA26:10,800,000–11,900,000) and in a second experiment the region at CFA26 was extended and a total of ≈3.4 Mb was targeted (CFA26: 8,500,000–11,900,000). Fragment libraries were prepared as described in Olsson et al. [61]. Sequence capture was performed using a 385K custom-designed probe array from Roche NimbleGen according to the manufacturer’s instructions. Captured enriched libraries were sequenced using Illumina sequencing technology. In the first experiment, six dogs: three brachycephalic (2 Boxer, 1 Pug) and three control breeds (1 Dachshund, 1 Welsh Corgi, 1 Basset Hound) were sequenced with a read length of 60bp (single end reads), using Genome Analyzer II. In the second experiment, four dogs: three brachycephalic+glioma (1 French Bulldog, 1 English Bulldog, 1 Boston Terrier) and one control (1 Dachshund) were sequenced with a read length of 100bp (paired end reads), using HiSeq 2000. Information about breed and health status for the individual dogs that were sequenced is shown in S3 Table. Obtained reads were mapped to CanFam 2.0 [18] using Burrows-Wheeler Aligner (BWA) [62]. SAMtools [63] was used for variant calling using mpileup format. Recommended setting for BWA reads -C50 was used as a coefficient to downgrade mapping quality, and–E to increase sensitivity. For coverage calculation the SEQscoring tool was used with SAMtools pileup format (S3 Table). Before variant calling, PCR duplicates were removed using the tool Picard (hosted by SAMtools). The presence of structural variants was investigated by comparing coverage for cases and controls using SEQscoring [38] and IGV [64].

### Evaluation of candidate variants

To select SNVs for genetic validation in additional dogs, we used SEQscoring [38] to score variants according to conservation (SiPhy constraint elements detected by the alignment of 29 eutherian mammals [39], phastCons [65] multiple alignment from UCSC of the human (hg17), mouse (mm6), rat (rn3), and the dog (canFam2) and a multiple alignment of 16 amniota vertebrates [66] from Ensembl release 56). SEQscoring [38] was also used to calculate pattern scores in order to rank the SNVs by association to phenotype. Pattern scores are based on pairwise comparison of all individuals for all variants, where variants are scored based on similarities and differences between cases and controls. Genotypes from a whole genome re-sequencing study of six pools of breeds were included as controls. The pools consisted of 12 wolves and 60 dogs from 14 other breeds as described in a study by Axelsson and colleagues [67]. When calculating the pattern score, we used three different sets of individuals. In the first set we evaluated the region that was the most associated with glioma (CFA26:9,176,012–10,799,999) with available samples (three glioma-diagnosed cases, one individual control and six control pools). In the second set we accounted for the possibility of a risk factor that might be fixed in breeds related to the ancestral Bulldog, and thus classified the breeds Boxer, French Bulldog, English Bulldog and Boston Terrier as cases. Four of the brachycephalic dogs had been diagnosed with glioma (S3 Table). The entire region was included in the third set (CFA26:8.5–11.9 Mb). A total of 56 SNVs (31 conserved and 25 non-conserved) were successfully genotyped using iPLEX Sequenom MassARRAY platform in a total of 168 dogs (34 cases and 134 controls) (S4 and S5 Tables). Association between SNV allele and phenotype was evaluated using PLINK [36] chi-square calculations.

### Collection of canine tissue samples

Primary tumor tissue was obtained at necropsy or from surgical biopsy of clinical cases presented to the VMTH. Necropsy samples were collected within 20 min after death. Control normal cerebral tissue was collected from contralateral cerebral hemispheres in tumor bearing dogs, and from neurologically normal dogs. All samples were snap-frozen in liquid nitrogen for storage. Samples of adjacent tumor tissue were paraffin-embedded and processed for histological analysis. All tumors were histologically classified by a board-certified pathologist according to the WHO classification of human tumors of the central nervous system [2]. All canine samples were obtained with their owner's consent, and in strict accordance with good animal practice, with study protocols approved by the Institutional Animal Care and Use Committee (IACUC) at UC Davis.

### Expression analysis in dogs

Total RNA was isolated from whole blood of control and affected dogs using QIAamp RNA Blood Mini Kit (QIAGEN, Valencia, CA). The optional on column DNase treatment was carried out to eliminate gDNA contamination. 100ng of total RNA was used for cDNA synthesis with the SuperScript III First-Strand Synthesis System for RT-PCR (Life technologies, Grand Island, NY). Primers for quantitative real-time PCR (qRT-PCR), for *PR2X7*, *CAMKK2* and *DENR* were designed using Primer3Plus [68] and are shown in S6 Table. Semi quantitative RT-PCR using AmpliTaq Gold® (Life Technologies, Grand Island, NY) was performed to confirm product size and sequence identity was confirmed by sequencing. PCR was performed in triplicates using the Rotor-Gene SYBR Green PCR Kit (QIAGEN). A 2 step cycle protocol (35 cycles; 95°C; Annealing- 15 seconds at 60°C; Extension- 90 seconds at 60°C; Final Melt curve) was carried out on the Rotor Gene Q real-time PCR instrument (QIAGEN). Each replicate containing 0.2ng template cDNA. All data were normalized to the housekeeping gene *B2M*, using published primer sequences [69]. Amplification efficiency and differences in takeoff values between affected and unaffected dogs were analyzed by REST2009 [70]. Box plots of calculated delta-delta CT values were generated using GraphPad Prism version 5 (GraphPad Software, La Jolla, CA).

### Western blotting canine tissues

Protein extraction and Western blots were carried out similarly as described before [16]. Briefly, tissues were lysed in RIPA buffer (Boston BioProducts, Inc., Worcester, MA) with 1X Halt protease and phosphatase inhibitors (Thermo Fisher Scientific, Inc., Rockford, IL) and proteins were quantified using a Coomassie protein assay reagent (Pierce/Thermo Fisher Scientific, Rockford, IL) 20ug of protein was heat denatured and resolved by SDS PAGE electrophoresis followed by transfer to a nitrocellulose membrane. Blots were blocked for 1 h and then incubated overnight with primary antibodies. Primary rabbit polyclonal antibodies used were anti-P2RX7 antibody recognizing amino acids 576–595 of rat P2X7R (1:500, APR-004 Alamone Labs, Jerusalem, Israel) and anti-actin antibody (1:10,000, A2066, Sigma-Aldrich, St Louis, MO). Blots were washed then incubated for 2 h with HRP-conjugated goat anti-rabbit IgG (1:5,000, 12–348, EMD Millipore, Temecula, CA). Blots were visualized with SuperSignal West Femto solution (Pierce/Thermo Fisher Scientific, Rockford, IL) and detection was performed with Vision Works LS digital capture software (UVP, Upland CA).

### Bioinformatics analysis of human expression data

One set of glioma and normal brain expression data was obtained from a study by Liu and colleagues [40] and can be found in the ArrayExpress database (GSE21354). Briefly, the dataset contains five samples of diffusely infiltrating astrocytic gliomas, four samples of ependymomas, five samples of oligodendrogliomas, and four samples of normal brain tissue. All 18 samples were hybridized on the Affymetrix GeneChip Human Genome U133 Plus 2.0. Raw CEL files where downloaded from ArrayExpress and processed in the Affymetrix Expression Console using the RMA algorithm for background correction, quantile normalization and probe summarization. Multiple transcripts/probes for the same gene were collapsed using the mean value. A second dataset of gene expression values in GBM and normal brain tissue was downloaded from TCGA (http://cancergenome.nih.gov/). Specifically, the data contained a total of 10 epileptic brain normal tissue samples processed in a single batch as well as 24 GBM tissue samples processed within the same batch. For these samples the downloaded data corresponded to level 3 gene expression as measured by the HT Human Genome U133A array. For further processing, the distribution of gene expression values within each sample was standardized using the z-score. One-way ANOVA analyses were employed to test for an overall significant difference between group means for each gene and two-sided Welch t-tests were employed to test for significant differences between the means of any pair of two groups.

### Human cell cultures

Human glioblastoma cell cultures were developed as follows: human GBM grade IV biopsies were obtained in accordance with the protocol approved by the Uppsala ethical review board (2007/353), and were graded by neuropathologist Irina Alafuzoff, Uppsala University Hospital, according to WHO guidelines. Tumor biopsies were minced (1 mm × 1 mm pieces) and digested by 1:1 ratio of Accutase (eBioscience, San Diego, CA)/TrypLE (Life technologies, Grand Island, NY) at 37°C for 15 min and triturated through 18g and 21g needles 5 times. Dissociated cells were resuspended in DMEM/F12 Glutamax (Life technologies, Grand Island, NY) and Neurobasal medium (Life technologies, Grand Island, NY) mixed 1:1 with addition of 1% B27 (Life technologies, Grand Island, NY), 0.5% N2 (Life technologies, Grand Island, NY), 1% penicillin/streptomycin (Sigma-Aldrich, St Louis, MO), 10 ng/ml EGF and FGF2 (Peprotech, Rocky Hill, NJ), and plated at 100,000 cells/ml. After primary sphere formation, spheres were seeded onto poly-ornithine/laminin-coated dishes and cultured as adherent cells as described in Pollard et al. [71].

### Differentiation assay for human glioma cells

Twenty thousand human glioma cells per well were seeded onto polyornithine-laminin coated glass coverslips in 6-well plates in maintenance medium (see above). The next day, medium was changed to differentiation medium, DMEM/F12 Glutamax: Neurobasal (ratio1:1) with 1% B27, 0.5% N2 supplement, 1% penicillin/streptomycin and with the addition of 5% FBS but no EGF or FGF-2. Medium was changed every 3–4 days. Seven days later, cells were lysed for RT-PCR.

### Quantitative RT-PCR of candidate genes in human cells

Cells were lysed, and total RNA was extracted using RNeasy Mini kit (Qiagen, Valencia, CA) according to instructions from the manufacturer. Genomic DNA was digested with RNase-free DNase I (Qiagen, Valencia, CA) throughout the RNA extraction process. Five hundred nanograms of RNA was transcribed into cDNA using iScript cDNA synthesis kit (BIO-RAD, Berkeley, CA). Quantitative RT-PCR was performed in triplicates with Ssofast EvaGreen Supermix kit (BIO-RAD, Berkeley, CA). A no-template negative control was included for each primer set and constantly found not to generate any PCR products. The primers used in this experiment are shown in S7 Table. The PCR products were loaded on 2% agarose gel and photographed using Molecular Imager gel-doc XR+imaging system (BIO-RAD, Berkeley, CA) and Image Lab software. For relative expression analysis, a comparative cycle threshold method (ΔΔCT) was used. Briefly, each gene of interest was first normalized against endogenous housekeeping control (β-actin), and then the normalized values were further normalized using the control sample.

### Western blot of human cells

Normal human frontal cortex lysate was purchased from Abcam. Human glioma cells were lysed in RIPA buffer (150 mM NaCl, 1% NP-40, 0.5% deoxycholate, 0.1% SDS, 50 mM Tris, pH7.5) containing 1% protease inhibitor (Roche Diagnostics). Lysate was centrifuged at 10,000g for 30min and supernatant was used for protein estimation using BCA protein assay kit (Thermo scientific, Rockford, IL) following the manufacturer’s instruction. Equal amount of protein was loaded onto 10% gel NuPAGE (Invitrogen). The protein was transferred to nitrocellulose membrane through iBlot gel transfer device (Invitrogen). Membrane was blocked in 5% milk in TBST for 1h, followed with overnight incubation with primary antibody in 4°C and incubation with secondary antibody for 1h in room temperature. Primary antibodies used were mouse anti-CAMKK2 antibody (1:200) from Abnova (Taipei, Taiwan), and mouse anti-beta-actin antibody (1:5000) from Sigma-Aldrich (St Louis, MO). Secondary antibody used was HRP goat anti-mouse IgG from GE healthcare (Little Chalfont, UK). Blot was visualized using Amersham ECL kit (GE healthcare, Little Chalfont, UK) and detection was performed with ImageQuant LAS 4000 (GE healthcare, Little Chalfont, UK).

## Supporting Information

S1 Fig Phylogenetic tree of dog breeds. A phylogenetic tree was constructed using SNP-data. Part of the tree is shown here supporting the closest relationship between the high-risk glioma brachycephalic dog breeds Boston Terrier, English Bulldog, Boxer and French Bulldog.
(DOCX)

S2 Fig GWAS for glioma excluding Boxers. Removing Boxers from the dataset retains the same distinct peak at CFA 26, with an even stronger association due to a reduction of stratification.
(PDF)

S3 Fig QQ-plot of GC values (Boxers excluded). There is a reasonably low stratification left after GC correction, where deviation from expected (black line) starts at p-value ≈2 -log10(GC) but deviates more sharply and considered significant from a p-value of ≈6.5 -log10(GC).
(PDF)

S4 Fig **Expression of *DENR* (A) and *P2RX7* (B) in human brain.** We found no significant difference for *DENR* or *P2RX7* expression using a subset of data from the Cancer Genome Atlas (TCGA) [11] consisting of mRNA expression data of surgical specimens from 24 glioblastoma patients, and 10 non-tumor control brains (epilepsy resections).
(TIF)

S1 Table Individual genotypes from glioma GWAS. The most associated SNPs from the glioma GWAS in red text. Homozygous SNPs for the two different alleles are colored red respective orange and heterozygous SNPs are colored blue.
(XLSX)

S2 Table P-values for the five most associated SNPs from GWAS. The five most associated SNPs from glioma GWAS, unadjusted and adjusted using genomic control (GC) calculated using PLINK software.
(DOCX)

S3 Table Coverage in target region. Sequenced reads were mapped to the whole genome reference of CanFam 2.0, and coverage/position (X) was calculated using SEQscoring checking every 20’th position in target region.
(DOCX)

S4 Table Location of evaluated SNVs. 56 SNVs identified in the re-sequencing data as putative candidates for glioma, were evaluated in a larger cohort of dogs. Annotation of homologues human genes in the UCSC browser noted at location. SNVs located within conserved elements according to any of the three different alignments of species described in Materials and Methods are marked with x.
(DOCX)

S5 Table Individual genotypes for evaluated variants. For each dog it is noted, if it has a brachycephalic (brachy) phenotype and/or has been diagnosed with glioma. Individual genotypes are shown for the two most associated SNVs to glioma at CFA26 (26.10893462 26.9722698) together with the one SNV (26.10984721) associated to glioma that caused a non-synonymous codon change. In addition genotypes for the identified structural variation is shown. The insertion/deletion at CFA 26 is marked with “5” for a single band of ≈5000 bp and “2” for the shorter variant of ≈2200 bp. Failed genotyping is marked with “0 0”.
(XLSX)

S6 Table Primers used for qRT-PCR dogs. (DOCX)

S7 Table Primers used for qRT-PCR humans. (DOCX)

S8 Table Allele frequencies for two risk alleles. The allele frequency per breed was calculated for the two most significant SNVs in the evaluation data set.
(DOCX)

S9 Table Confirming genotyping of candidate mutations. Additional genotyping was performed in order to confirm candidate variants and calculate odds ratios for 6 breeds at risk that are segregating at the associated locus.
(XLSX)
