# Supplementary figures and images for: Utilizing the Dog Genome in the Search for Novel Candidate Genes Involved in Glioma Development—Genome Wide Association Mapping followed by Targeted Massive Parallel Sequencing Identifies a Strongly Associated Locus
Source: PLoS Genet. 2016 May 12;12(5):e1006000. doi: 10.1371/journal.pgen.1006000 (PMC4865040; doi:10.1371/journal.pgen.1006000)

**
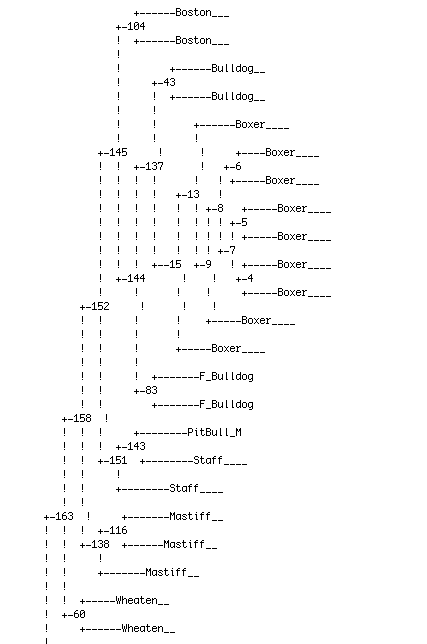
**

Supplement: S1 Fig — A phylogenetic tree was constructed using SNP-data. Part of the tree is shown here supporting the closest relationship between the high-risk glioma brachycephalic dog breeds Boston Terrier, English Bulldog, Boxer and French Bulldog. (DOCX) [file pgen.1006000.s001.docx]

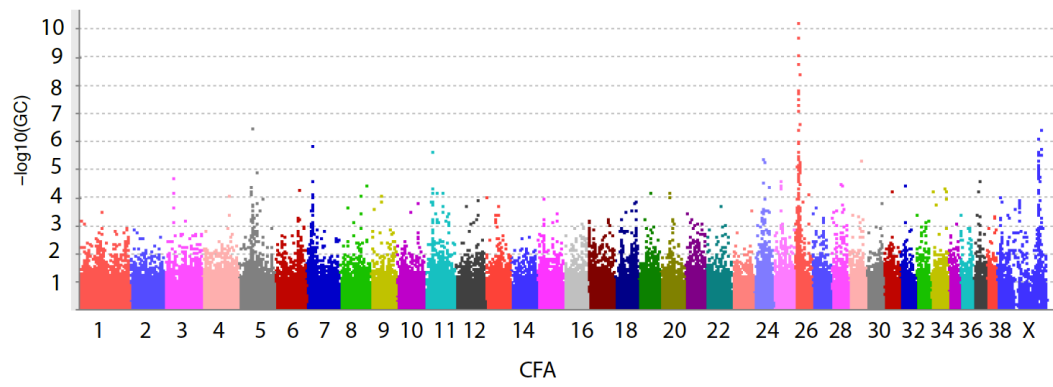

Supplement: S2 Fig — Removing Boxers from the dataset retains the same distinct peak at CFA 26, with an even stronger association due to a reduction of stratification. (PDF) [file pgen.1006000.s002.pdf]

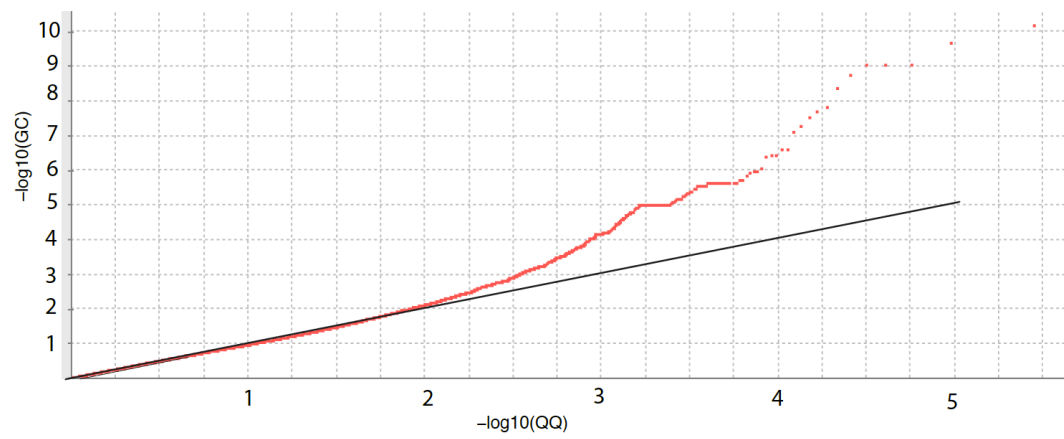

Supplement: S3 Fig — There is a reasonably low stratification left after GC correction, where deviation from expected (black line) starts at p-value ≈2 -log10(GC) but deviates more sharply and considered significant from a p-value of ≈6.5 -log10(GC). (PDF) [file pgen.1006000.s003.pdf]

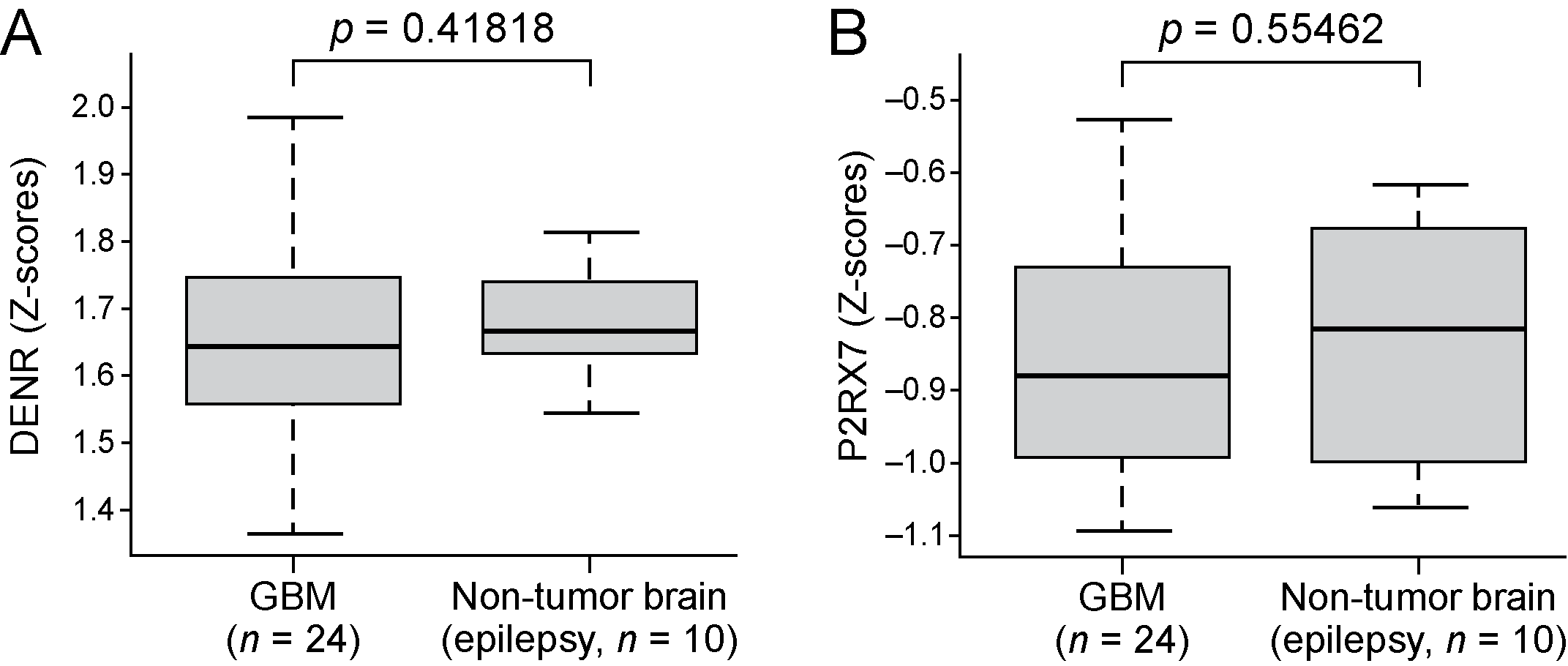

Supplement: S4 Fig — Expression of DENR (A) and P2RX7 (B) in human brain. We found no significant difference for DENR or P2RX7 expression using a subset of data from the Cancer Genome Atlas (TCGA) [11] consisting of mRNA expression data of surgical specimens from 24 glioblastoma patients, and 10 non-tumor control brains (epilepsy resections). (TIF) [file pgen.1006000.s004.tif]
