# Supplementary material for: Utilizing the Dog Genome in the Search for Novel Candidate Genes Involved in Glioma Development—Genome Wide Association Mapping followed by Targeted Massive Parallel Sequencing Identifies a Strongly Associated Locus
Source: PLoS Genet. 2016 May 12;12(5):e1006000. doi: 10.1371/journal.pgen.1006000 (PMC4865040; doi:10.1371/journal.pgen.1006000)
Supplement: S2 Table — The five most associated SNPs from glioma GWAS, unadjusted and adjusted using genomic control (GC) calculated using PLINK software. (DOCX) [file pgen.1006000.s006.docx]

| **CHR** | **SNP** | **UNADJ** | **GC** |
| --- | --- | --- | --- |
| 26 | chr26.9780187 | 5.473e-38 | 2.774e-08 |
| 26 | chr26.9580770 | 7.794e-37 | 4.601e-08 |
| 26 | chr26. 10744458 | 4.41e-35 | 9.931e-08 |
| 26 | chr26. 10839073 | 4.41e-35 | 9.931e-08 |
| 26 | chr26. 10864137 | 4.41e-35 | 9.931e-08 |
