# Supplementary material for: Utilizing the Dog Genome in the Search for Novel Candidate Genes Involved in Glioma Development—Genome Wide Association Mapping followed by Targeted Massive Parallel Sequencing Identifies a Strongly Associated Locus
Source: PLoS Genet. 2016 May 12;12(5):e1006000. doi: 10.1371/journal.pgen.1006000 (PMC4865040; doi:10.1371/journal.pgen.1006000)
Supplement: S3 Table — Sequenced reads were mapped to the whole genome reference of CanFam 2.0, and coverage/position (X) was calculated using SEQscoring checking every 20’th position in target region. (DOCX) [file pgen.1006000.s007.docx]

| **ID** | **Breed** | **Status** | **Read size (bp)** | **SE/PE^*^** | **≥ 5X (%)** | **Mean X** |
| --- | --- | --- | --- | --- | --- | --- |
| S1 | Dachshund | healthy, non-brachycephalic | 60 | SE | 88 | 23 |
| S2 | Pug | healthy, brachycephalic | 60 | SE | 90 | 31 |
| S3 | Welsh Corgi | healthy, non-brachycephalic | 60 | SE | 85 | 17 |
| S4 | Boxer | healthy, brachycephalic | 60 | SE | 81 | 14 |
| S5 | Basset Hound | healthy, non-brachycephalic | 60 | SE | 82 | 15 |
| S6 | Boxer | glioma, brachycephalic | 60 | SE | 78 | 12 |
| S7 | English Bulldog | glioma, brachycephalic | 100 | PE | 94 | 42 |
| S8 | French Bulldog | glioma, brachycephalic | 100 | PE | 96 | 68 |
| S9 | Boston Terrier | glioma, brachycephalic | 100 | PE | 96 | 68 |
| S10 | Dachshund | healthy, non-brachycephalic | 100 | PE | 88 | 24 |

* Single-end reads (SE), paired-end reads (PE).
