# Supplementary material for: Utilizing the Dog Genome in the Search for Novel Candidate Genes Involved in Glioma Development—Genome Wide Association Mapping followed by Targeted Massive Parallel Sequencing Identifies a Strongly Associated Locus
Source: PLoS Genet. 2016 May 12;12(5):e1006000. doi: 10.1371/journal.pgen.1006000 (PMC4865040; doi:10.1371/journal.pgen.1006000)
Supplement: S4 Table — 56 SNVs identified in the re-sequencing data as putative candidates for glioma, were evaluated in a larger cohort of dogs. Annotation of homologues human genes in the UCSC browser noted at location. SNVs located within conserved elements according to any of the three different alignments of species described in Materials and Methods are marked with x. (DOCX) [file pgen.1006000.s008.docx]

| CFA | **Position** | **Conserved** | **Location** |
| --- | --- | --- | --- |
| 26 | 10234713 | x | intronic MLXIP |
| 26 | 10461927 | x | intronic SETD1B |
| 26 | 10487040 | x | LOC338799 non-coding RNA |
| 26 | 10576572 | x | intergenic |
| 26 | 10694694 | x | intronic KDM2B |
| 26 | 10770781 |  | intronic RNF34 |
| 26 | 10773721 | x | exonic RNF34, synonymous |
| 26 | 10774388 | x | intronic RNF34 |
| 26 | 10782685 | x | intronic RNF34 |
| 26 | 10893462 |  | intronic CAMKK2 |
| 26 | 10940449 |  | intergenic |
| 26 | 10958779 |  | intergenic |
| 26 | 10969340 |  | intronic P2RX7 |
| 26 | 10983106 |  | intronic P2RX7 |
| 26 | 10984721 | x | **exonic P2RX7, F->L** |
| 26 | 10987069 |  | intronic P2RX7 |
| 26 | 10990192 |  | intronic P2RX7 |
| 26 | 10990331 |  | intronic P2RX7 |
| 26 | 11031222 | x | intergenic |
| 26 | 11031273 | x | intergenic |
| 26 | 11041971 | x | intronic IFT81 |
| 26 | 11050456 |  | intronic IFT81 |
| 26 | 11051152 |  | intronic IFT81 |
| 26 | 11071041 |  | intronic IFT81 |
| 26 | 11099291 |  | intronic IFT81 |
| 26 | 11102491 | x | intronic IFT81 |
| 26 | 11106982 | x | intronic IFT81 |
| 26 | 11117351 |  | intronic IFT81 |
| 26 | 11118474 |  | intronic IFT81 |
| 26 | 11127749 | x | intergenic |
| 26 | 11145402 |  | intergenic |
| 26 | 11153442 |  | intergenic |
| 26 | 11155459 |  | intergenic |
| 26 | 11174086 | x | intronic ATP2A2 |
| 26 | 11174153 | x | intronic ATP2A2 |
| 26 | 11249843 | x | intronic ANAPC7 |
| 26 | 11380341 | x | intronic PPTC7 |
| 26 | 11438085 | x | exonic TCTN1, synonymous |
| 26 | 11456369 | x | exonic HVCN1, synonymous |
| 26 | 11462366 |  | intronic HVCN1 |
| 26 | 11483745 |  | intergenic |
| 26 | 8793655 |  | intronic DNAH10 |
| 26 | 8847141 |  | intronic DNAH10 |
| 26 | 8994769 | x | splice site C12orf38 |
| 26 | 9147724 |  | intergenic |
| 26 | 9361634 | x | intronic MPHOSPH9 |
| 26 | 9428185 |  | intergenic |
| 26 | 9717490 | x | exonic 3' UTR DENR |
| 26 | 9720111 | x | intronic DENR |
| 26 | 9721116 | x | intronic DENR |
| 26 | 9722698 | x | intronic DENR |
| 26 | 9723848 |  | intronic DENR |
| 26 | 9727925 | x | intronic DENR |
| 26 | 9764257 | x | intronic DENR |
| 26 | 9843958 | x | exonic KNTC1, synonymous |
| 26 | 9894200 | x | intronic RSRC2 |
