# Supplementary material for: Utilizing the Dog Genome in the Search for Novel Candidate Genes Involved in Glioma Development—Genome Wide Association Mapping followed by Targeted Massive Parallel Sequencing Identifies a Strongly Associated Locus
Source: PLoS Genet. 2016 May 12;12(5):e1006000. doi: 10.1371/journal.pgen.1006000 (PMC4865040; doi:10.1371/journal.pgen.1006000)
Supplement: S6 Table — (DOCX) [file pgen.1006000.s010.docx]

| Gene | Forward 5’ 🡪 3’ | Conc (µM) | *T*m (°C) | Reverse 5’ 🡪 3’ | Conc (µM) | *T*m (°C) | Product length (bp) |
| --- | --- | --- | --- | --- | --- | --- | --- |
| P2RX7 | ACACTGCGGATTACACCTTCC | 4 | 62.6 | GTTGGCCTTCTGTCTTGAGAAAG | 3 | 62.8 | 100 |
| CAMKK2 | AATCGCCATCCTCAAGAAGC | 2 | 60.4 | TGACCAGCTCAAACACCATG | 2 | 60.4 | 120 |
| DENR | GGGGAAGAGGAATTGCATGTG | 2 | 62.6 | TCAGCAGCCATCTCACAAAC | 3 | 60.4 | 90 |
| B2M | TCCTCATCCTCCTCGCT | 1 | 61.2 | TTCTCTGCTGGGTGTCG | 1 | 61.2 | 85 |
