# Supplementary material for: Utilizing the Dog Genome in the Search for Novel Candidate Genes Involved in Glioma Development—Genome Wide Association Mapping followed by Targeted Massive Parallel Sequencing Identifies a Strongly Associated Locus
Source: PLoS Genet. 2016 May 12;12(5):e1006000. doi: 10.1371/journal.pgen.1006000 (PMC4865040; doi:10.1371/journal.pgen.1006000)
Supplement: S7 Table — (DOCX) [file pgen.1006000.s011.docx]

| **Gene** | **Species** | **Sequence** |
| --- | --- | --- |
| CAMKK2 | human | forward: 5'-CGTATGCTGGACAAGAACCC-3'  reverse: 5'-TCTCATAAGGACACAAAGCC-3' |
| DENR | human | purchased from qiagen |
| P2RX7 | human | forward: 5'-TATGAGACGAACAAAGTCACTCG-3'  reverse: 5'-GCAAAGCAAACGTAGGAAAAGAT-3' |
