# Supplementary material for: Utilizing the Dog Genome in the Search for Novel Candidate Genes Involved in Glioma Development—Genome Wide Association Mapping followed by Targeted Massive Parallel Sequencing Identifies a Strongly Associated Locus
Source: PLoS Genet. 2016 May 12;12(5):e1006000. doi: 10.1371/journal.pgen.1006000 (PMC4865040; doi:10.1371/journal.pgen.1006000)
Supplement: S8 Table — The allele frequency per breed was calculated for the two most significant SNVs in the evaluation data set. (DOCX) [file pgen.1006000.s012.docx]

| Breeds | Cases | Controls | CFA26:10,893,462 | CFA26:9,722,698 |
| --- | --- | --- | --- | --- |
| Boxer | 9 | 5 | 1.00 | 1.00 |
| English Bulldog | 2 | 8 | 1.00 | 1.00 |
| Japanese Chin |  | 2 | 0.00 | 0.00 |
| Pekingese |  | 5 | 0.10 | 0.10 |
| Pug |  | 9 | 0.00 | 0.00 |
| Boston Terrier | 2 | 7 | 0.93 | 0.93 |
| Australien Cattledog | 1 | 4 | 0.00 | 0.00 |
| Australien Shepherd | 2 | 8 | 0.19 | 0.06 |
| Nova Scotia Duck Tolling Retriever |  | 13 | 0.00 | 0.19 |
| Jack Russel Terrier | 1 | 10 | 0.20 | 0.10 |
| Labrador Retriever | 6 | 9 | 0.28 | 0.22 |
| Weimaraner |  | 5 | 0.00 | 0.00 |
| Rhodesian Ridgeback | 1 | 7 | 0.14 | 0.00 |
| Soft Coated Wheaten Terrier | 1 | 3 | 0.50 | 0.00 |
| West Highland White Terrier | 1 | 7 | 0.14 | 0.14 |
| Mastiff | 2 | 7 | 0.14 | 0.36 |
| Whippet |  | 1 | 0.00 | 0.00 |
| Pit Bull Mix | 3 |  |  |  |
| Pit Bull Terrier |  | 10 | 0.50 | 0.45 |
| Staffordshire Terrier | 2 | 8 | 0.69 | 0.50 |
| Keeshond | 1 | 6 | 0.08 | 0.00 |
|  | 34 | 134 |  |  |
